# Adaptation to elastic loads and BMI robot controls during rat locomotion examined with point-process GLMs

**DOI:** 10.3389/fnsys.2015.00062

**Published:** 2015-04-28

**Authors:** Weiguo Song, Iahn Cajigas, Emery N. Brown, Simon F. Giszter

**Affiliations:** ^1^Department of Neurobiology and Anatomy, Drexel University College of Medicine, Drexel UniversityPhiladelphia, PA, USA; ^2^Brain and Cognitive Sciences, Massachusetts Institute of TechnologyCambridge, MA, USA; ^3^Institute for Medical Engineering and Science, Massachusetts Institute of TechnologyCambridge, MA, USA; ^4^Department of Anesthesia, Critical Care and Pain Medicine, Harvard Medical School, Massachusetts General HospitalBoston, MA, USA; ^5^School of Biomedical Engineering, Drexel UniversityPhiladelphia, PA, USA

**Keywords:** augmenting BMI, elastic field, point-process general linear model, exoskeleton robot model system, motor adaptation

## Abstract

Currently little is known about how a mechanically coupled BMI system's actions are integrated into ongoing body dynamics. We tested a locomotor task augmented with a BMI system driving a robot mechanically interacting with a rat under three conditions: control locomotion (BL), “simple elastic load” (E) and “BMI with elastic load” (BMI/E). The effect of the BMI was to allow compensation of the elastic load as a function of the neural drive. Neurons recorded here were close to one another in cortex, all within a 200 micron diameter horizontal distance of one another. The interactions of these close assemblies of neurons may differ from those among neurons at longer distances in BMI tasks and thus are important to explore. A point process generalized linear model (GLM), was used to examine connectivity at two different binning timescales (1 ms vs. 10 ms). We used GLM models to fit non-Poisson neural dynamics solely using other neurons' prior neural activity as covariates. Models at different timescales were compared based on Kolmogorov-Smirnov (KS) goodness-of-fit and parsimony. About 15% of cells with non-Poisson firing were well fitted with the neuron-to-neuron models alone. More such cells were fitted at the 1 ms binning than 10 ms. Positive connection parameters (“excitation” ~70%) exceeded negative parameters (“inhibition” ~30%). Significant connectivity changes in the GLM determined networks of well-fitted neurons occurred between the conditions. However, a common core of connections comprising at least ~15% of connections persisted between any two of the three conditions. Significantly almost twice as many connections were in common between the two load conditions (~27%), compared to between either load condition and the baseline. This local point process GLM identified neural correlation structure and the changes seen across task conditions in the rats in this neural subset may be intrinsic to cortex or due to feedback and input reorganization in adaptation.

## Introduction

Brain Machine Interfaces (BMIs) hold the potential to provide functions to spinal-injured patients (Hochberg et al., 2006) or to augment normal motor functions in novel ways. The operation of BMIs depends on reliable neural decoding during ongoing behaviors and on a subject's adaptation of activity to achieve function across differing ongoing tasks. To date, most efforts in BMI have worked in mechanically isolated contexts and concentrated on highly-trained manipulation tasks of the forelimb (Chapin et al., 1999; Hochberg et al., 2006; Moritz et al., 2008; Velliste et al., 2008). They have largely neglected locomotion or its augmentation using invasive single unit methods, with notable exceptions (Fitzsimmons et al., 2009; Song et al., 2009; Manohar et al., 2012; Alam et al., 2014). Further, a future BMI application of lower limb control would likely require the user to manage on-line motor adaptations and integrate the lower limb BMI control with their whole body control.

The effect on neural modulation of BMI integration into the body scheme has not been thoroughly investigated. The stability of cortical neural responses remains controversial. Some data showed that cells can be functionally stable and BMI systems maintain performance across days with the same decoder (Serruya et al., 2003; Greenberg and Wilson, 2004; Chestek et al., 2007). However, significant neural plasticity was also reported in non-BMI force-field adaptation experiments and in BMI systems exposed to a given decoder across days (Li et al., 2001; Carmena et al., 2005; Jackson et al., 2006; Rokni et al., 2007; Zacksenhouse et al., 2007; Jarosiewicz et al., 2008; Ganguly and Carmena, 2009; Song and Giszter, 2011). Even when responses become fairly stable, and switching among BMI controls are achieved easily, for example after over-training, issues will remain in integrating and adapting the BMI controls to new tasks and mechanical contexts in activities of daily living. Conceivably, highly-trained BMI designs with complicated decoding algorithms may impede the use of the intrinsic plasticity of the brain to adapt and improve the BMI control in dynamic environments. Current efforts to develop adaptive decoders must balance their potential advantages against the potential for conflict with the user's own adaptation. An alternative strategy may be to provide simpler interfaces (Moritz et al., 2008), and allow the brain's plasticity and processing power to adapt and incorporate the novel actions.

Using a relatively simple interface strategy to generate a neural driven force from the robot, like Fetz and colleagues (Moritz et al., 2008), we examined the use of the BMI in this dynamic environment. Rats were exposed to three conditions during treadmill locomotion:(a) control, (b) simple elastic loading (E), and (c) BMI with elastic load (BMI/E) in which BMI lifting control was made available in parallel with the elastic load used in (b). Previously, we found that cells could modulate their firing patterns differently during each adaptation in different experimental conditions (Song and Giszter, 2011).

To understand the function of neuronal circuits and systems, it is essential to characterize the connections between individual neurons (Brown et al., 2004; Shigeyoshi, 2008; Stevenson and Körding, 2010) and their dynamics across tasks or condition changes. Correlations of cells relate to network connectivity and common inputs and processing. The cross-correlation, coherence and joint peri-stimulus time histogram (JPSTH) methods have been used for pairs of cells (Gerstein and Perkel, 1969; Jarvis and Mitra, 2001; Schneidman et al., 2006; Shigeyoshi, 2008). To characterize the strengths and any dynamics of the connections between neurons, variants of information based methods have been used in computational neuroscience for analyzing spiking neural systems (Strong et al., 1998). However, in many brain areas, each neuron receives input from a large population, and the generalized linear model (GLM) provides another framework to examine connectivity based on the point process representation of the spike trains (Brown et al., 2002; Truccolo et al., 2005; Acharya et al., 2008; Stevenson et al., 2008; Héliot et al., 2010). The GLM attempts to predict a neuron's firing patterns based on its own spikes and the spikes of other neurons, and on external inputs. By combining a KS statistical analysis for the goodness of fit in each model, it provides a powerful tool for neural network functional connectivity analysis (Brown et al., 2002; Truccolo et al., 2005; Acharya et al., 2008; Kositsky et al., 2009; Gerhard et al., 2011). By utilizing a point process framework and GLM, we examined the neural dynamics at the functional network level.

We examined “functional connectivity” which we here define as an analysis and estimation of how firing of each neuron appears to influence each other neuron in a given data set, given the data collected and included in the analysis, with such influences expressed in the most parsimonious way possible. This represents a compact description of firing pattern correlations and apparent influences on one another in the observed data. Because the neural activity is incompletely observed, it is necessarily limited in relation to anatomy and actual circuit structure. Results showed significant differences in network organization as estimated by the GLM analysis of functional connectivity under different conditions, with some fraction of “core connectivity” in the GLM models, and the observed firing pattern features, preserved across the trials.

## Methods and materials

### Surgical procedure and neural recording

Six adult Sprague-Dawley rats, weighing 250 ~ 300 g, were used in these experiments. Care and treatment of the animals conformed to protocols approved by the University Laboratory Animal Resources and Institutional Animal Care and Use Committee of the College of Medicine of Drexel University. Methods are published in Song and Giszter (2011). The detailed procedures for pelvic and cortical implantations have also been described previously (Song and Giszter, 2011). For short, after anesthesia using KXA (63 mg/Kg Ketamine, 6 mg/Kg Xylazine, 0.05 mg/Kg Acepromazine), rats were implanted with a pelvic orthosis, which allowed a PHANTOM robot (Sensable Devices) to apply forces directly to the skeleton. Tetrode arrays, which consisted of 6 tetrodes positioned around the neck of an etched spear-shaped fine tungsten rod, were implanted stereotactically in the hindlimb / trunk area of the motor cortex (2.0 mm lateral of the midline, 1.6 mm caudal to bregma and 1.0–1.5 mm in depth). Recording sites of all electrodes in a rat were within from 50 to 200 microns of one another in the cortex using our probe design. Intracortical microstimulation of rat M1 in this recorded region generates hindlimb/trunk movements in all rats tested. At the end of the final recording session, strong electrolytic lesions were made and brain slices were cut in 40 μm sections to validate electrode placement. Neural data were recorded using a Cerebus system (Cyberkinetics, Inc. / Blackrock systems) after animals recovered from surgery. Neural signals were band-pass filtered (300–7.5 KHz) and digitized at a sampling frequency of 30 kHz. Spikes were detected online using thresholding. The detected spikes could be automatically classified on-line after setting the templates of each waveform. The on-line sorted spikes were used in a real time “BMI with elastic load” control. The controller and real-time OS code were custom laboratory written code (available on request). They implemented the algorithms in Equation (1) below, with the parameters noted there. The detected spike trains, as well as all the thresholded spike waveforms, were also saved for off-line analysis. Multiple single units were isolated off-line using Off-line Sorter (Plexon Neurotech Com.) after noise removal by cluster cutting method (Shoham et al., 2003). This analysis uses the offline sorted data. Up to 24 channels of neural activity at a time in a single array could be recorded, and 1 or 2 individual cells from most wires of the tetrodes could be recorded; around 15–38 well isolated single units were used in each trial.

### Robot controls and training protocols

The robotic system employed and the training protocol used have been described in detail previously (Song and Giszter, 2011). Briefly, different force fields (linear, non-linear, isotropic, anisotropic, piecewise discontinuities etc.) could be applied to the pelvis of the rat using our custom control software. This was achieved through an implanted orthosis, directly interacting with the skeleton using the orthosis attached robot. Robot control was updated at a frequency of 1 KHz, and these controls were synchronized and coupled with neural recordings. Neural data recorded could be used in the robot control scheme after a delay of under 3 ms from spike occurence. In elastic load trials (*abbrevation* ***E***), unidirectional (vertical forces only, i.e., radially anisotropic) elastic fields were used. Elastic loading (downward) fields *F*_*e*_ were applied in condition E by setting an equilibrium point on the horizontal equilibrium plane of the elastic field around 12.5 mm under the rat's normal pelvic height with a field stiffness of 44 N/m, which gives around 15% of the body weight. In trials termed here “BMI with elastic load” (abbrev. BMI/E), a neural driven lifting elastic force *F*_*n*_ was combined with the above simple elastic force *F*_*e*_, and both forces were combined and applied simultaneously. The neural driven elastic force was calculated according to the equation

(1) $$F_{n}=K_{n}\times N_{af}\times \left(X-X_{n0}\right),$$

where *N*_*af*_ was aggregate firing rate in the prior 100 ms window, *K*_*n*_ = 80 *N/m* was the stiffness, *X*_*n*0_ = 55 mm was the equilibrium point, and *X* the current vertical position of the pelvis with respect to the horizontal equilibrium plane. This gave the rat in BMI/E trials a means to offset the elastic load *F*_*e*_ using neural activity *N*_*af*_ to modulate an opposing force *F*_*n*_. Experiments except where noted comprised one or more iterations of 3 conditions: 2 min of baseline (BL) with no loading, followed by 2 min of elastic load (E) in which the rat experienced *F*_*e*_, and then 2 min of BMI + load (BMI/E) in which the rat had access to the BMI driven field *F*_*n*_ and could potentially offset *F*_*e*_.

Rats were trained to walk on a treadmill at a speed ranging from 0.1 to 0.15 m/s depending on the normal preferred walking speed of individual rats. One daily experimental session used in analysis below was divided into three trials as described above: (i) the BL (baseline) trial, in which rats walked on treadmill without any force; (ii) the E (simple elastic load) trial; (iii) the BMI/E (BMI with elastic load) trial. Each session thus consisted of three 2-min trials of treadmill walking (~120 steps) with 5 min intervals of rest occurred between each trial.

### Neural network functional connectivity analysis by using GLM model

We follow Stevenson et al. (2009) and Okatan et al. (2005) in defining functional connectivity: we seek to characterize how each neuron appears to influence each other neuron in the data sets in the most parsimonious way possible. The neural modulation of single cells that was observed during BMI usage and motor adaptation observed in our experimental paradigm was previously reported (Song and Giszter, 2011).

**Figure 1:**
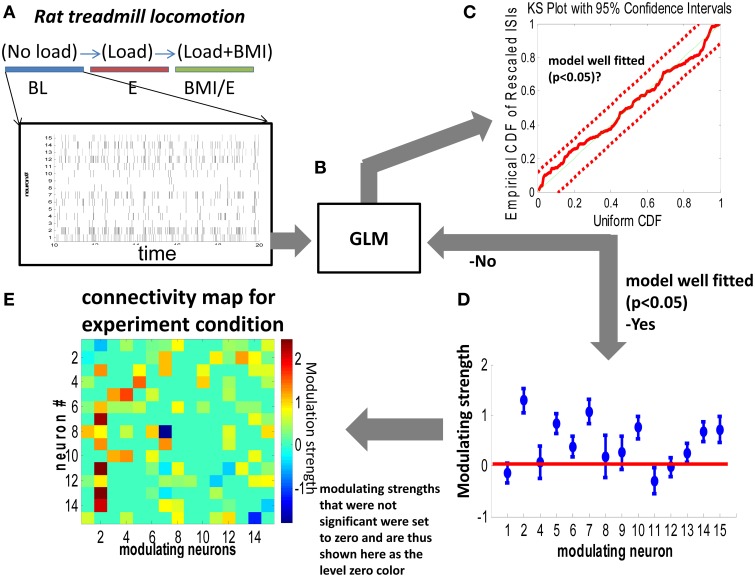
**Generalized linear model (GLM) based network connectivity analysis. (A,B)** The conditional intensity function of each neuron was modeled with GLM by using a single step spiking history of the whole population of neurons over two different timescales (1 ms vs. 10 ms). **(C)** A Kolmogorov Smirnov (KS) criteria was used to test the goodness of fit of the model. According to the time rescaling theorem for point processes, the rescaled spike times under the true conditional intensity function would be independent, identically distributed, exponential random variables with unit rate. **(D)** For neurons which showed a significant nonzero contribution in the GLM model, the nonzero modeling strength was used to construct an element in the connectivity matrix (map) in **(E)**. Parameters whose confidence interval crossed zero (the horizontal red line) in **(D)** were deemed not significantly different from zero and were set to zero in the connectivity maps in **(E)**.

The procedure for functional connectivity analysis was as shown in Figure 1. Briefly, neural spiking activity is modeled as a point process which is completely characterized by its conditional intensity function (Daley and Vere-Jones, 2003) defined as

(2) $$\lambda \left(t\;|\;H_{t}\right)=\underset{\;\Delta \to 0}{\lim}\frac{P(N\left(t+\Delta \right)-N\left(t\right)=1\;|\;H_{t})}{\Delta }$$

where *H*_*t*_ is all the history information from 0 up to time *t*, and *N*(*t*) is a counting process containing the sum of all the spikes up to time *t*. The conditional intensity function can be considered a generalization of the “rate” for a homogenous Poisson process. Under the restriction that λ(*t*|*H*_*t*_)Δ follows a distribution in the exponential family, the GLM framework can be used to fit statistical models for the conditional intensity function (McCullagh and Nelder, 1989). Goodness of fit can be assessed by Kolmogorov-Smirnov test (KS-test) after the time rescaling theorem (Brown et al., 2002).

The conditional intensity function of the *i*th spiking neuron was modeled with the spiking history of neighbor cells either at a short timescale (1 ms, *L* = 1) or long timescale (10 ms, *L* = 10) as Equation (3),

(3) $$\lambda_{i}\left(t_{k}\;|\;H_{t}\right)=\exp\left(\mu_{i}+\sum_{\begin{array}{l} j\;=\;1\\ j\neq i \end{array}}^{J}\;\beta_{ij}\cdot △N_{j}\left(t_{k}\right)\right)$$

where *J* is the total number of neurons recorded in the current trial; μ_*i*_ is the baseline level of firing rate; Δ*N*_*j*_(*t*_*k*_) = *N*_*j*_ (*t*_*k*_) − *N*_*j*_(*t*_*k*−*L*_) is the number of spikes of the *j*th neuron between time *t*_*k*−*L*_ and *t*_*k*_, *L* = 1 or 10 for the short and long timescale model respectively, and β_*ij*_ is the regression coefficient to be estimated representing the strength of effect between the firing of the *j*th neuron in the short or long timescale and the current firing rate of the *i*th neuron. We term the β_*ij*_ as the functional connectivity coefficient of neuron *j* to neuron *i*. Note that with this definition, β_*ij*_ ≠ β_*ji*_ in general and both that both short and long timescale models have the same number of parameters. We define the functional connectivity map, **β**, as the matrix containing β_*ij*_ in the *i*th row and *j*th column. The structure of **β** is one measure of the connections between neurons or a representation of the functional network between the neurons.

The activity of each neuron was modeled by its neighboring neurons' immediately preceding history within a 10 ms window (type A in Equation 3) and in a small number of test models by its own history (type B, self-history included - i.e., remove the *j* ≠ *i* constraint in Equation 3). The Type B model (with neural self history) was evaluated in 2 rats. Models with the self history inclusion on the timescales tested here differed little in their network neighbor interactions. They showed significant overlap of network connections with the similar networks identified and estimated without self history. However, they generally showed some reduction of external network connections (22% in BL, 11% in E, and 15% in BMI, when using 1 ms time bins) with increasing history parameters but also a decreasing quality metric (see below). Here we present data from the network models without self-history, consistent with our quality metric used (see below).

### Statistical methods

After GLM fitting (glmfit, MATLAB) for each neuron, a Kolmogorov-Smirnov (KS) test was applied to test the goodness of fitting. If the fitting was within the 95% confidence interval, we classified the neuron as well fitted. Then, the significant parameters that were nonzero (*p* < 0.05) were used to construct the functional connectivity map as in Figure 1.

In order to test the functional connectivity map changes between two conditions, and their significance, the functional connectivity map identified and estimated in each was converted to a binary functional connectivity matrix by setting the *significant* nonzero elements to one and the others to zero as in **Figure 4**. Connection density was defined as the percentage of significant connected links, in relation to the total possible links and used as a criterion for analysis and comparison of model structures and conditions. In a first pass analysis we modeled all recorded neurons as stationary Poisson processes to test for non-Poisson behavior. In a second pass analysis (type II models), we modeled those neurons whose firing was not fitted by a mean rate parameter, and thus non-Poisson by our test criterion used for model fit assessment (time rescaling theorem/Kolmogorov Smirnov tests with *p* < 0.05). Such cells could not be well-predicted simply by using a mean rate parameter (type I model) and were thus firing based on locomotion variables or one another. The number of such non-Poisson cells found differed in the data sets, varying based on binning. We found such “non-Poisson patterned” cells using both 1 ms and 10 ms binning. In total 573 neurons were analyzed. Cell numbers that were non-Poisson were as follows: 1 ms binning: 287 (BL), 328 (E), 303(BMI/E); 10 ms: 348 (BL), 368 (E), 361 (BMI/E). The first pass analysis was equivalent to an assumption of the possible presence of neither locomotion task information nor neural interaction information in the cell firing. Cells not fitted in this way would need a more complex fitting, which could further improve conditional intensity function fit and KS statistic if modeled correctly, using neural or locomotion variable effects. The second pass analysis we used examined neural interactions alone. Effectively, we assumed that the first pass Poisson fitted cells were unrelated to the motor tasks, and were simply noise sources in the cortical network, though potentially important predictors of other neural firing. We ignored the external covariate effects, focusing on neural correlation alone. We compared GLM model structures to capture the non-Poisson population of neurons at a short timescale using 1 ms binning and at a longer timescale using 10 ms binning based on the parsimonious selection of GLM fitting. We sought models that captured the most neurons with the best levels of individual neuron parameter parsimony. To assess this model quality we calculated a model parameter Q,

(4) Q=n/N

where *n* is the percentage of neurons with non-Poisson firing that were well fitted using the model, *N* is the square root of the significant connection density (number of possible connections growing as the square of neurons recorded). High Q values indicate good parsimonious fitting of the (non-Poisson) activity in the network, and the sparsest possible connectivity description of firing. If two models had the same numbers of well-predicted neurons total in a data set but one fitting had higher Q (i.e., fewer connections/ parameters required) it was considered a better model form. If two models fitted different numbers of neurons in a data set, but the model fitting of larger number of neurons had a much lower Q this would indicate a more complex individual neural functional connectivity was necessary in the model to capture the greater number of neurons and achieve fitting. When the activity of each neuron was modeled from its neighboring simultaneous firing neurons, but not using self history and recursive self connections, the overall parsimony and Q value obtained was significantly lower, so we only present data using the history effects of neighbor neurons not self history (Equation 3).

From the binary functional connectivity matrix of significant parameters found using models with the best Q, we could then test the significance of functional connectivity maps changes. We did by using a binomial distribution test. That is, by assuming the probability of keeping or changing the original link had the same chance, then if the probability of the pattern of change observed was less than 0.05, then a significant change had occurred. In practice this statistical test was likely unnecessary, since we found that only about 15–27% of model connections persisted across different conditions in an animal. Nonparametric signed rank tests (signrank, MATLAB) were used to compare the significance of such individual changes and differences across sessions and rats. All the tests were set to a significance level of 0.05. All data analysis was performed using custom software written in MATLAB. A suite of refined and related MATLAB tools including these is freely available (Cajigas et al., 2012).

## Results

In total 21 robot and BMI interaction sessions were collected in 6 normal rats that were implanted with multi-electrode recording probes. Rats performed treadmill locomotion in three conditions: (1) unloaded baseline (BL), (2) a robot driven elastic load condition (E) in which rats experienced a vertical elastic load, and (3) a robot driven elastic load and BMI condition (BMI/E), see Figure 1A. In the last condition the rat could in principle offset elastic load using neural activity. In BMI/E the recorded neural activity of the rat regulated the stiffness of a second “BMI elastic field.” This could potentially be employed to offset the elastic load, as was described in Song and Giszter (2011). We have reported previously that rats do quite quickly begin to adjust and to offset the load (Song and Giszter, 2011). We sought to assess changes in functional connectivity as rats proceeded from BL to E to BMI/E conditions. In order to do this we compared functional connections in GLM fitted networks for the data (Figures 1A–E) in each successive test condition. We thus separately fitted each condition. 21 fitted networks were here obtained and compared for each condition (BL, E, and BMI/E) using GLM models. We tested fits of the GLM models at two different binning timescales: 1 ms and 10 ms. In total 573 neurons were analyzed in the 21 sessions. Cell numbers that were non-Poisson and supported network connection modeling were as follows: 1 ms binning: 287 (BL), 328 (E), 303 (BMI/E); 10 ms: 348 (BL), 368 (E), 361 (BMI/E). In addition to these sessions, in control experiments we tested repetitions of the baseline, and repetitions of the full sequence of tests in a rat, to assess the connection and network drift (gradual network functional connection changes within a condition), relative to the measures of change we saw between conditions.

### Network fitting procedures and network characteristics

**Figure 2:**
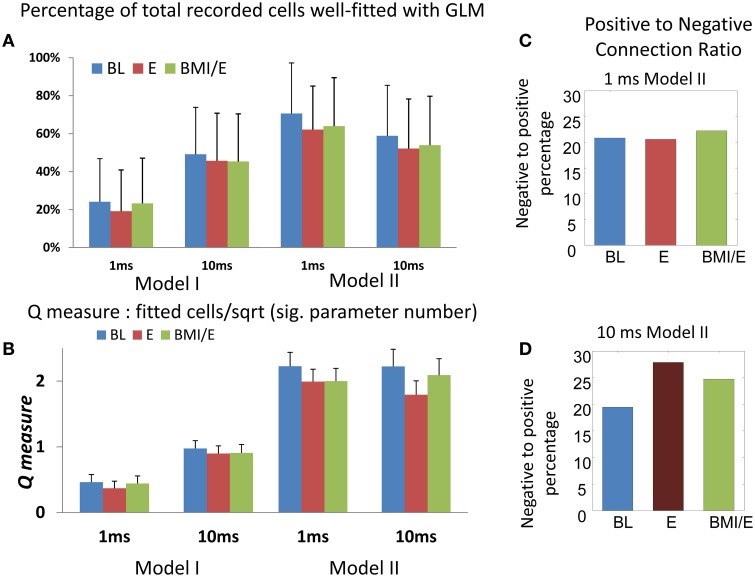
**Numbers of cells fitted with GLM model and Q-measure**. (**A**) Percentage of total cells from the whole population (*n* = 573) fitted in each model and adaptation condition. The model II with 1 ms timescale had the highest capture of cells and best Q (indicated with the red dotted box). Note that despite good overall fits, only about 15% of neurons with firing interval statistics not captured by a mean rate parameter (non-Poisson firing) were well-predicted in the GLM networks here. More total and non-Poisson cells were fitted in the E condition than in the other two conditions (BL or BMI/E) at both short timescale (1 ms binning) and long timescale (10 ms binning). **(B)** The Q-measure. This was defined as the ratio of the percentage of neurons with non-Poisson firing that were well-predicted using the model, to the square root of the significant connection density required. Coupled with **(A)** the Q-measure analysis favors the type II models at the 1 ms binning scale. The error bars indicates mean ± S.E. **(C)** The percentage of significant negative parameters as a portion of significant positive modulation parameters in the 1 ms bin model II is shown in each task condition. **(D)** The percentage of significant negative parameters as a portion of significant positive modulation parameters in the 10 ms bin model II is shown in each task condition. The number of significant negative interactions was lower than positive ones in each network, while there was no significant difference between the different adaptation conditions for each model. Overall, in the 10 ms binned model II fits, the number of negatively modulating interactions increased. Abbrev. BL baseline, E elastic load, BMI/E brain machine interface and elastic load.

We first modeled each neuron in each task condition (BL, E, and BMI/E) by using a 1 step spiking history of the ensemble neurons using either short timescale (1 ms bin) or longer timescale (10 ms bin) steps. Type I models ignored interactions, simply fitting cells independently as point processes to discover non-Poisson pattern cells. Type II models used cell interactions. We found that about 60% of recorded cells were well fitted at the short timescale. However, the number of well-predicted non-Poisson firing cells was not high. About 15% of such non-Poisson pattern cells were well-predicted using the short timescale. Significantly less cells were well-predicted in the long timescale, matching the total cell fitting patterns. The numbers of total and non-Poisson firing pattern cells that were well fitted at each timescale were not significantly different between the three different adaptation conditions. We used a measure Q of fitting complexity to compare the models and binnings (see Methods). The numbers of cells that were well-predicted and the Q statistic in both the shorter timescale (1 ms) and longer timescale (10 ms) analyses are shown in Figure 2. The 10 ms binned fits were almost as good as the 1 ms model fits. Although a simple 1 step history linear filter is presented here, we also tested type I and type II models with basis function filters similar to Truccolo (Truccolo et al., 2010), and tested models with a 10 ms (five basis function) history in two rats. There was no significant improvement in the number of cells which could be well-predicted using KS-criteria, when compared to using the 1 ms binning used here. Thus, we kept the 1 step history window in our subsequent modeling. We present the analyses only for the simpler 1 ms bin and 10 ms history type II model. In the 1 ms bin short timescale, the GLM models captured the binary spike train behaviors, where temporal coding might feature, while the 10 ms bin long timescale GLM model corresponds more to rate coding. More cells could be well fitted using the short timescale models than with the long timescale models. This suggests that with 1 ms binning, the individual cells predicted (or “influenced”) the short term information of the neighboring cells better (more) than was seen on the long timescale. Thus, the better-fitted functional connectivity network structures examined here using 1 ms binning likely represent short timescale processes.

We also examined the fitted networks' significant parameters to see if numbers of excitatory or inhibitory types of connections were dominant, and the average strengths of each type of connection. In each network, we found that the number of significant negative interactions (“inhibition”) was lower than the number of positive ones (“excitation”) constituting about on fifth on average (Figures 2C,D). When we compared these connection numbers for the models between experimental conditions, we found that there was no significant difference in the positive/negative parameter balances in models among the different task conditions. However, the number of negatively modulating interactions in the models using 10 ms binning and timescale were increased overall compared to 1 ms binning and timescale (Figures 2C,D).

### Network stability in continuously repeated trials of a condition, and repetitions of conditions after delays and condition switches

**Figure 3:**
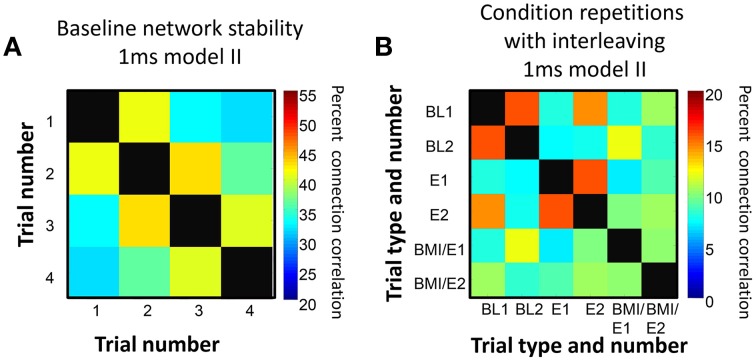
**Connection persistence within condition. (A)** Repeated baseline recordings with 5 min pauses between them showed more than 30% of connections were preserved from the first to last recording. However, over the repetitions there was a slow drift in connection pattern. The matrix of percentage of common connections, and the color map scale are shown. On the diagonal, the black squares indicate 100% correlation (self to self). **(B)** Repetitions of the basic experiment used, with successive trials of BL, E, BMI/E in a rat. The matrix of common connection percentages is organized with BL1 and BL2 grouped, E1 and E2 grouped, and BMI/E1 and BMI/E2 grouped but these were separated in time in the experiment. The repetitions of each condition are generally more similar to one another than to other conditions. The level of within condition similarity is not consistent with random assortment of the matrix elements and their variance. On the diagonal, the black squares indicate 100% correlation (self to self). Note also that the color scale range in **A** and **B** differ.

In control trials, we found that in successive repetitions of the baseline conditions, the fitted networks showed some connection variability but that overall more than 30% of connections were preserved over the entire series of repetitions, and around 40% or more similarity was found between tests of networks adjacent in time (Figure 3A). Similarly, we found that networks fitted to repetitions of each condition after other interleaved conditions showed more connection similarity with each other (Figure 3B) than with the networks fitted to different conditions that were adjacent in time, and this difference was significant under an assumption of random variations (e.g., for data in Figure 3B, one-tailed *t*-test *p* < 0.05).

### Network changes across different adaptation conditions

**Figure 4:**
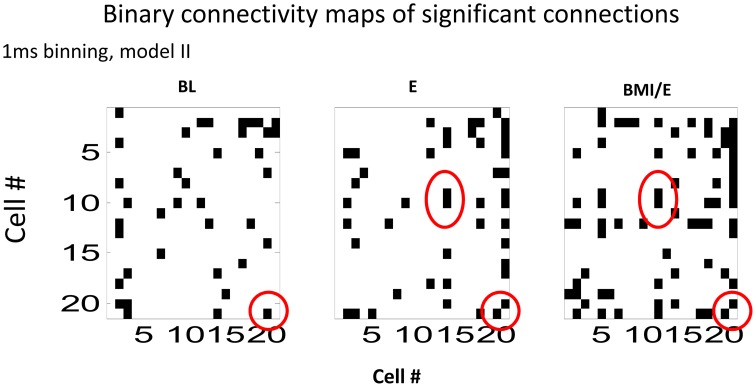
**Binary connectivity maps from the GLM fits in a typical session (from the same session as is shown in Figure 5)**. Significant connections are shown in black. The networks showed sparse connectivity. The connection density was higher for the 1 ms bin time scale that is shown (1 ms binning). Examples of a few connections found in common in the GLM fits in each condition, are circled in red. These show consistency in modeling of some connections across all the conditions. Abbrevations: BL, baseline; E, elastic load; BMI/E, brain machine interface and elastic load.

**Figure 5:**
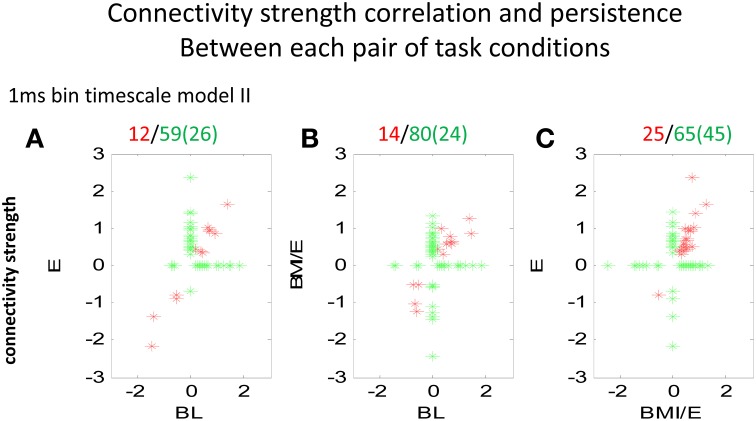
**Comparison of connectivity strengths between two conditions modeled at the 1 ms binned timescale**. Each point marker in the figure denotes a significant parameter and connectivity strength from a pair of neurons, related between two conditions (**A** BL vs. E, **B** BL vs. BMI/E, and **C** E vs. BMI/E). The red markers denote that the connection between the neurons was significantly different from zero under both the two tested conditions, while the green marker denotes the connectivity of the neurons was only significantly different from zero under one condition (BL, E or BMI/E). The numbers in parentheses indicate number of cells on abscissa. Some of the network connectivity estimated with the GLM models shifted drastically, and much connectivity was unique to a given condition. However, there were also connections that persisted across conditions (red). These persistent connection strengths clearly also correlated. There were more connections in common between E and BMI/E in **C**, than in either **A** or in **B** when these were correlated to BL. Abbrevations: BL, baseline; E, elastic load; BMI/E, brain machine interface and elastic load.

**Figure 6:**
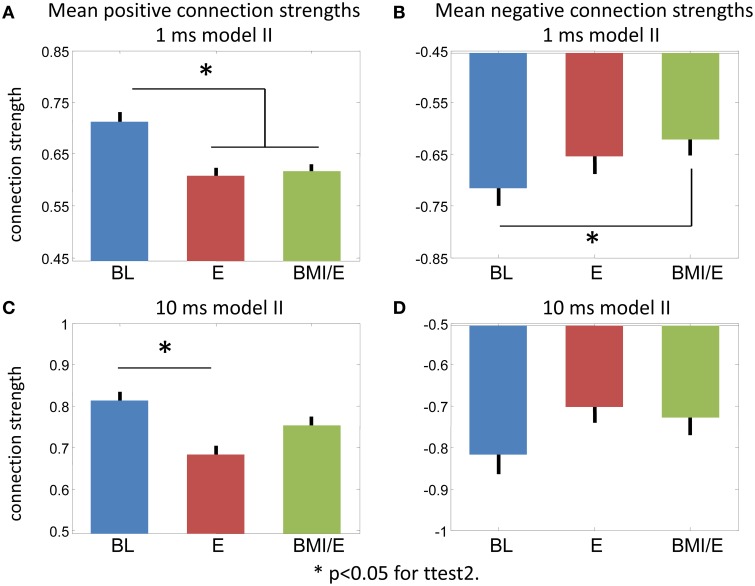
**Connection strength changes. (A,B)** Model II 1 ms bin fitting. **(A)** Positive connections. **(B)** Negative connections. **(C,D)** Model II 10 ms bin fitting. (**C**) Positive connections. **(D)** Negative connections. ^*^ Significant difference in a two-tailed *t*-test, *p* < 0.05 (MATLAB ttest2).

We next analyzed the model connectivity changes occuring between the three adaptation conditions when they followed in close succession in a complete experiment series. Our rationale was that although the numbers of cells, which could be well-predicted by either type I or type II GLM models were not significantly different among each adaptation task condition, the network structure might change or reorganize in these different contexts. Thus, we looked in detail at how the fitted network statistics changed in the transitions between the different task conditions, compared across conditions, and at the different binning timescales. Both the binary connection maps (Figure 4) and the functional connectivity strengths (Figures 5, 6) showed significant differences at the two different model timescales (1 ms, and 10 ms). These also differed among the three adaptation conditions tested (BL, E, and BMI/E). Some fraction of the connections persisted across different task conditions (Figure 4, circled examples). Connections that persisted in common between the three adaptation conditions remained correlated in strengths (Figure 5 red points in each plot), however other patterns of functional connectivity often changed significantly. The binary functional connectivity maps showed distinct patterns depending on timescales analyzed, as seen in Figure 4. Generally, we found that in the longer 10 ms binned data most connections were unique and many fewer were shared with the other adaptation conditions (not shown). We therefore focused accordingly on the short time scale 1 ms binned models (1 ms Type II Models) see Figures 4, 5. We showed previously that rats in BMI/E offset load while preserving more normal pelvic height compared to load alone (Song and Giszter, 2011). When rats experiencing applied loads in BMI/E were able to significantly offset these with the BMI, the network increased the number of connections during simple elastic load and BMI with elastic load conditions compared to baseline (e.g., see Figures 4, 5). Further, significantly more of (almost double the number of) the connections persisted in the transition from Elastic load (E) to BMI (BMI/E) (~27% in Figure 5C) compared to those that seen were in common between Baseline and Elastic load (~16% in Figure 5A) or Baseline and BMI (~15% in Figure 5B). This statistically significant difference in common or shared functional connectivity was biased to positive connections between the two load conditions (Figure 5C, one red negative connection in common out of 25 total common), but instead included higher fractions of negative parameter connections when examining connections that were in common with baseline patterns of firing (Figure 5A, 4/12 negative, and Figure 5B, 4/14).

While the numbers of significant connections increased in the E and BMI/E conditions, the mean parameter strengths also altered. Figure 6 shows the average positive and negative connection strengths tended in 1 ms and 10 ms models. In keeping with the other results the 10 ms binning results had weaker significance, only differing in positive connection strength between BL and BMI/E. In the 1 ms models, all connection strengths tended to decrease in the loading conditions compared to baseline. These changes were significant for the positive connections in both load conditions in the 1 ms models, and for negative connections compared to BMI/E.

**Figure 7:**
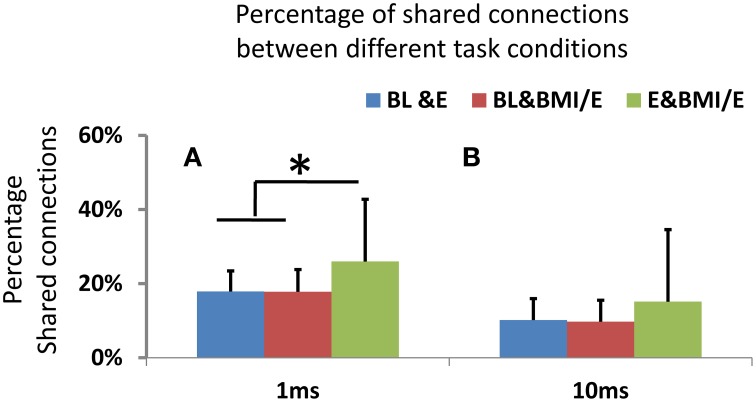
**(A)** Shared connections between networks obtained in two conditions. On either 1 ms or 10 ms binned timescales, the networks showed significant differences in connectivity between two conditions (BL vs. E, BL vs. BMI/E and E vs. BMI/E) using binomial tests. However, with 1 ms binning there was a significantly higher percentage of shared connection between E and BMI/E than that between any other two conditions (BL vs. E and BL vs. BMI/E, indicated by ^*^, two-tailed *t*-test, *p* < 0.05, MATLAB ttest2). **(B)** For the 10 ms binning there was no significant difference in the percentage of shared connection between any paired conditions, and percentages of shared connections were significantly lower than in the 1 ms binned GLM models.

By using functional connection density measures and the shared significant parameters as functional connectivity links or edges in a network graph, we compared the network structure changes. We found the number of common connections was significantly decreased in 10 ms timescale binned models compared to 1 ms binned (signed rank test, *p* < 0.05, MATLAB signrank), as in Figure 7. These differences held across baseline condition and elastic load condition as well as BMI with elastic load. All the networks built from the GLM models showed low numbers of shared connections (15–27%) and significant differences (assessed by signed rank tests, *p* < 0.05) between any two conditions. However, there were significantly higher percentages of shared connections between the two load adaptation conditions of E and BMI/E than between either of the load with baseline condition shared connections (BL vs. E and BL vs. BMI/E) (Figure 7A). The percentage of shared connections between any two compared conditions was also higher in 1 ms binning than in 10 ms binning Figure 7B. These differences among conditions could not be explained by drift over time in BL e.g., in Figure 3A. This suggests that the active cortical circuitry may in part share a similar common structure when rats adapt under simple elastic load and under BMI with elastic load, and may also indicate that apart from rate encoding (analyzing in long timescale), short term temporal coding (binary spike timing) might capture some information regarding these changes on a short timescale.

In conclusion, the fitted GLM model networks of cells here showed significantly different connection maps between each task condition potentially indicating significant changes in network dynamics with task conditions. A core of between 15 and 27% of network connections were in common between the individual conditions and over time, but this common core differed in extent between different task condition pairs, with baseline having less in common with the other two (loaded) conditions.

## Discussion

Using the methods here we previously demonstrated that rats could adapt their locomotion under a simple elastic load and use a BMI to offset elastic load effectively (Song and Giszter, 2011). GLM methods allow us to move beyond our prior analyses of coarse firing rate in this paradigm, and to add point process assessments of functional connectivity. The differences in BMI adaptation compared to the simple load in this paradigm involve alterations in firing patterns and rate based correlation relationships among neurons, but not average firing rates compared with elastic loading (Song and Giszter, 2011). We previously also observed changes in the number of force-related neurons, their peak firing and burst activity timing in the step cycle (Song and Giszter, 2011). Here we focus on neuron-neuron interactions at the point process level among neurons all collected within a range of from 30 to 200 microns of each other in each rats, with the recording electrode sites around layer V of motor cortex. These spatially close neurons may potentially have different connectivity from spatially further separated neurons. As we have noted above the functional connectivity we uncovered here represents the most parsimonious account of apparent neural influences among one another based on the observed neurons and firing. Because not all neurons are observed the account is necessarily incomplete and may infer Granger type causal links where none exist in the complete biology. However, subject to these limitations the functional connectivity may give descriptive insights into aspects of network operation. By using a GLM approach here, our data analysis has shown some features of cortical network dynamics occuring at different timescales during these adaptation processes. The neural network in hindlimb/trunk cortex of rats as assessed here in a subset of well-predicted neurons was extensively reorganized in terms of GLM parameters during adaptation to different elastic load force fields applied in locomotion. The networks shared more common connections between elastic load and BMI with elastic load condition than between any other condition pairs. More of the functional network connections identified were preserved during transitions from elastic load (E) to BMI with elastic load (BMI/E) conditions, than from baseline (BL) to elastic load (E). However, only about 15% of the total population of non-Poisson firing cells were well-predicted by our models here, which were built without reference to the external covariates, and relied only on strong neural firing pattern interactions. Internal network functional connectivity and the network based firing predictions that are presented here thus represent an account of a small fraction of the patterned activity. The models thus also indicate a high degree of independence of much of the non-Poisson pattern firing cells from one another from the GLM modeling perspective, consistent with classical rate coding models and other frameworks (e.g., Sanger, 2010, 2011, 2014). Likely the strongest predictive relationships of the firing of the 85% of unfitted non-Poisson cells will be with external covariates and tuning to feedback or motor drive. Nonetheless, the internal relationships revealed here in well-predicted cells provide some insights into the adaptation processes and dynamics in augmenting BMIs (see Omar et al., 2011). Unfortunately, low firing rates for the GLM point process models preclude a confident analysis of the activity changes within a condition as was possible with other methods in Song and Giszter (2011). The relations of the local activity relations here to overall function of the network remain to be determined.

It should be noted that caution should be taken when interpreting the results from model dynamics and differences here. As noted, the model did not consider external covariates, which might have made a strong contribution to the models. This was especially likely during elastic load conditions, and some if not most of the non-Poisson firing pattern cells that were not well fitted with neural interactions alone can be expected to be captured with inclusion of these. Whether and how the sparse connections identified here are correlated with the overall network states across tasks still needed to be tested. On the other hand, as the model here used spiking history of the ensemble of recorded cells, any effects of the potential external covariates on the well-predicted subset of cells analyzed here might be captured by its being filtered and incorporated into the history effects of connected neighboring neurons in the ensemble. The lagged parameters of GLM models of cells here generally had a more significant effect on a current cells' firing than any immediate (1 ms) synchronization to other firing cells consistent with causal interactions but no proof of such. Similiarly, it should be made clear that the connections or functional connectivity maps constructed here were derived only from “apparent pattern” among neural spikes. There might be no anatomical connectivity representing the apparent connections in the cortical network.

The differences between open loop BMIs, BMIs with feedback, physically connected BMIs and differences between BMIs for proximal locomotion vs. distal reaching are not well understood (Acharya et al., 2008; Héliot et al., 2010). However, these different effects may become crucial as various feedback based BMIs are introduced and the range and scope of available BMI prosthetic devices increases (Moritz et al., 2008; Velliste et al., 2008; Fitzsimmons et al., 2009; Héliot et al., 2010; Song and Giszter, 2011). Recent studies showed that cells in the primary motor cortex demonstrated learning related plasticity during force field adaptation (Li et al., 2001). A stable relation between neural activity and behavior can clearly be established in well trained animals (Serruya et al., 2003; Greenberg and Wilson, 2004; Chestek et al., 2007). Moritz et al. demonstrated that individual cells can be rate modulated to control Functional Electrical Stimulation (Moritz et al., 2008). Variations of neural firing patterns in such differing adaptation processes are not well understood at this point. For example, patterns might differ between novel voluntary tasks and more vegetative “built-in” or evolutionarily older tasks, such as locomotion. The rats adapted to load (E) and adapted differently to load with BMI (BMI/E). The identified *functional* cortical network parameters, as identified from firing observed here, also clearly adapted, and reorganized differently between conditions. Previously we found the distribution of average firing rate from the whole population was not significantly different among conditions (Song and Giszter, 2011). The numbers of significantly connected cells here were not significantly different across conditions in these models either. However, the numbers of common connections over time were higher in BMI with elastic load than baseline, and the network connection density even decreased during elastic loading alone when the rat adapted from the first half trial to the second half trial. Zacksenhouse et al. showed that there are firing rate changes in the early sessions with other BMI experiments (Zacksenhouse et al., 2007), and we found here that 70–85% of the sparse network connections of well-predicted cells reorganized depending on the context. For elastic load locomotion, the connection density actually decreased here, while in the BMI with elastic load locomotion condition, the network as modeled kept the same connection density. Similar results were obtained examining more standard long timescale correlations of rate in our earlier study (Song and Giszter, 2011). These data might indicate that rats need a longer time to achieve a stable and perhaps more sparse network state during BMI/E adaptation and conditioning when they were tested in our experimental framework, compared to the simpler elastic loaded locomotion. This evolving relationship between cortical firing effects, network changes, and feedback and relationships with external covariates is an area of active investigation using this framework.

When animals adapt their locomotion to new environments, initially cortex becomes more activated and engaged. Engaging a BMI in this period appears to alter and at least briefly stabilize the changes involved in the adaptation of neural network structure. Despite the small role of cortex in the normal locomotion of rats, cortex can be engaged in a BMI augmenting locomotion during this adaptation. Our data thus supports the idea that activity normally involved in monitoring, or simply correlated with and not normally driving a behavior in an animal, can nonetheless be engaged through motor adaptation processes to drive behavior in a BMI. Neuron-neuron correlation metrics estimated here provide one set of measures on the changes in neural dynamics on short adaptation time scales that can be extended to high practice use of BMIs and load adaptation over time, to examine transitions from naive user of an augmenting BMI to an expert practiced user. Understanding the patterns of these adaptive changes better may be essential in order to provide prostheses that can integrate seamlessly with normal motor adaptations and feedback effects across the many contexts encountered in activities of daily living.

### Conflict of interest statement

The authors declare that the research was conducted in the absence of any commercial or financial relationships that could be construed as a potential conflict of interest.
